# Kaempferol induces ROS-dependent apoptosis in pancreatic cancer cells via TGM2-mediated Akt/mTOR signaling

**DOI:** 10.1186/s12885-021-08158-z

**Published:** 2021-04-12

**Authors:** Fengjiao Wang, Lai Wang, Chao Qu, Lianyu Chen, Yawen Geng, Chienshan Cheng, Shulin Yu, Dan Wang, Lina Yang, Zhiqiang Meng, Zhen Chen

**Affiliations:** 1 Department of Integrative Oncology, Fudan University Shanghai Cancer Center, 270 Dong An Road, Shanghai, 200032 China; 2 Department of Oncology, Shanghai Medical College, Fudan University, Shanghai, 200032 China; 3 Cancer Center, Tenth People’s Hospital of Tongji University, Shanghai, 200072 China; 4 Cancer Institutes, Fudan University, Shanghai, 200032 China; 5 Department of Genetics and Cell Biology, Qingdao University Medical College, Qingdao, 266071 China

**Keywords:** Pancreatic cancer, Kaempferol, Tissue transglutaminase, ROS, Apoptosis, Biomarker

## Abstract

**Background:**

Kaempferol, a natural flavonoid, exhibits anticancer properties by scavenging reactive oxygen species (ROS). However, increasing evidence has demonstrated that, under certain conditions, kaempferol can inhibit tumor growth by upregulating ROS levels. In this study, we aimed to investigate whether kaempferol effectively suppresses pancreatic cancer through upregulation of ROS, and to explore the underlying molecular mechanism.

**Methods:**

PANC-1 and Mia PaCa-2 cells were exposed to different concentrations of kaempferol. Cell proliferation and colony formation were evaluated by CCK-8 and colony formation assays. Flow cytometry was performed to assess the ROS levels and cell apoptosis. The mRNA sequencing and KEGG enrichment analysis were performed to identify differentially expressed genes and to reveal significantly enriched signaling pathways in response to kaempferol treatment. Based on biological analysis, we hypothesized that tissue transglutaminase (TGM2) gene was an essential target for kaempferol to induce ROS-related apoptosis in pancreatic cancer. TGM2 was overexpressed by lentivirus vector to verify the effect of TGM2 on the ROS-associated apoptotic signaling pathway. Western blot and qRT-PCR were used to determine the protein and mRNA levels, respectively. The prognostic value of TGM2 was analyzed by Gene Expression Profiling Interactive Analysis (GEPIA) tools based on public data from the TCGA database.

**Results:**

Kaempferol effectively suppressed pancreatic cancer in vitro and in vivo. Kaempferol promoted apoptosis in vitro by increasing ROS generation, which was involved in Akt/mTOR signaling. TGM2 levels were significantly increased in PDAC tissues compared with normal tissues, and high TGM2 expression was positively correlated with poor prognosis in pancreatic cancer patients. Decreased TGM2 mRNA and protein levels were observed in the cells after treatment with kaempferol. Additionally, TGM2 overexpression downregulated ROS production and inhibited the abovementioned apoptotic signaling pathway.

**Conclusions:**

Kaempferol induces ROS-dependent apoptosis in pancreatic cancer cells via TGM2-mediated Akt/mTOR signaling, and TGM2 may represent a promising prognostic biomarker for pancreatic cancer.

## Background

Pancreatic cancer is the third most lethal malignancy, with associated mortality rates that continue to increase [1]. To date, surgery remains the only curative therapy for patients with pancreatic cancer. However, only 10–20% of patients have access to curative resection due to the notoriously difficult diagnosis [2]. Moreover, there have been few improvements in first- and second-line palliative therapies or progress in increasing survival with adjuvant treatment in pancreatic cancer [3]. Therefore, it is imperative to identify biomarkers for the early identification of novel, effective therapies for pancreatic cancer.

Numerous factors are involved in the instability and pathogenesis of pancreatic cancer, such as proinflammatory cytokines, oxidative stress and microenvironment-associated proteins. Among them, oxidative stress is commonly referred to as a process in which reactive oxygen species (ROS) produced by cellular metabolism are coupled with ROS scavenging by antioxidative mechanisms that prevent potential ROS-induced cytotoxicity and maintain cell survival [4]. In recent years, it has become apparent that ROS are implicated in the regulation of biological and physiological processes [5, 6]. Elevated ROS production is generally thought to be protumorigenic, leading to DNA, protein and lipid damage, increasing adaptations to hypoxia and promoting genetic instability and tumorigenesis [7, 8]. However, the roles of ROS in pancreatic cancer were shown to function as a double-edged sword that either facilitates cancer progression or dramatically promotes cell death [9, 10]. Evidence has demonstrated that cell apoptosis and senescence occur at toxic levels of ROS [11]. Given the dual roles of ROS, eliminating or increasing ROS levels may be a potentially effective cancer therapy, although realizing the potential of this approach has remained challenging.

Flavonoids are secondary plant metabolites that include kaempferol, luteolin and apigenin and are widely known to attenuate tissue damage or fibrosis through their antioxidant and anti-inflammatory activities [12, 13]. Kaempferol was recently reported to have ROS-mediated anticancer properties, including the inhibition of cell proliferation, cell cycle arrest, the induction of apoptosis and the suppression of cell invasion and migration, in diverse cancers, such as colorectal cancer, breast cancer, melanoma and hepatoma [14–17]. For example, kaempferol triggers ROS-induced apoptosis in human breast cancer through the JNK signaling pathway [16] and potentiates apoptosis in human hepatoma cells through the p53-inducible gene 3 (PIG3)-regulated mitochondrial apoptotic pathway [17]. However, it remains unclear how kaempferol alters ROS levels (upregulation or downregulation), as do the underlying mechanisms by which kaempferol inhibits pancreatic cancer.

In the present study, we investigated the anticancer activities of kaempferol on pancreatic cancer, the results of which showed that kaempferol promotes the potentiation of apoptosis and the inhibition of cell proliferation. Moreover, tissue transglutaminase (TGM2) overexpression via lentiviral vector-mediated transfection reversed the kaempferol-mediated induction of apoptosis. Collectively, our results demonstrated that kaempferol induces apoptosis in pancreatic cancer cells via ROS-mediated Akt/mTOR pathway inactivation, and that pharmacological inhibition of TGM2 by kaempferol in vitro and in vivo may provide a novel treatment option for the treatment of pancreatic cancer.

## Materials and methods

### Cells lines and reagents

The human pancreatic cancer cell lines Mia PaCa-2 and PANC-1 were obtained from the American Type Culture Collection (ATCC, USA). Cell lines were cultured in Dulbecco’s modified Eagle’s medium (DMEM) supplemented with 10% fetal bovine serum (FBS), 100 μg/ml penicillin and 100 μg/ml streptomycin in a humidified incubator at 37 °C under an atmosphere with 5% CO_2_. Kaempferol powder (C_15_H_10_O_6_, molecular weight: 286.24, purity > 99.0%) was purchased from Sigma-Aldrich (Shanghai, China). Kaempferol is a lipophilic polyphenol that is soluble in organic solvents. A 150 mM kaempferol stock solution was prepared in dimethyl sulfoxide (DMSO) and stored at − 20 °C. N-Acetyl-L-cysteine (NAC) was obtained from Beyotime (Shanghai, China).

### Establishment of TGM2-overexpressing cell lines

We used a lentiviral vector-mediated transfection method to overexpress TGM2 in Mia PaCa-2 and PANC-1 cells. The coding sequence of TGM2 was inserted into the Ubi-MCS-3FLAG-SV40-EGFP-IRES-puromycin lentiviral vector (GV358; GENE, Shanghai, China). The lentivirus TGM2-overexpressing constructs were co-transfected with pHelper 1.0 and pHelper 2.0 vectors at a ratio of 4:3:2 into HEK293T cells.

### Cell viability assay

A Cell Counting Kit-8 (CCK-8; DOJINDO, Shanghai, China) was used to measure cell viability. In brief, Mia PaCa-2 and PANC-1 cell suspensions (1 × 10^4^ cells/ml) were seeded in 96-well plates with 200 μl of DMEM and preincubated for 24 h. After adherence, the cells were treated with different doses of kaempferol (0, 2.5, 10, 25,100, 250 and 1000 μmol/L) and then incubated at 37 °C for an additional 48 h. After the indicated incubation times, the cells were incubated with a CCK-8 DMEM solution (10 μl of CCK-8 in 100 μl of DMEM) for 2 h. Then, the absorbance at 450 nm was measured using a microplate reader. The half maximal inhibitory concentration (IC50) values, which indicate 50% inhibition of cell viability, were determined by generating proliferation inhibition curves. Concentrations lower than the IC50 values were used in subsequent experiments.

### Cell apoptosis assay

Cell apoptosis was evaluated by flow cytometry by staining cells with FITC Annexin V in combination with propidium iodide (PI) or 7-amino-actinomycin (7-AAD) (BD Bioscience, San Diego, CA, USA) according to the manufacturer’s recommendations.

### Colony formation assay

Suspended pancreatic cells were seeded in 6-well plates at a density of 500 cells/well. Then, the cells were treated with 0, 25 or 50 μM kaempferol after attachment for 48 h. After 2 weeks of cultivation, cells that formed visible colonies were washed with phosphate-buffered saline (PBS) three times and fixed with 4% paraformaldehyde for 20 min for further staining with 0.1% crystal violet. Cell colonies containing more than 50 cells were counted using a light microscope.

### Western blotting

Cells were treated with kaempferol, washed with ice-cold PBS and then lysed with RIPA buffer supplemented with phenylmethanesulfonyl fluoride (PMSF) and phosphatase inhibitor. Cell lysates were centrifuged at 15,000 rpm and 4 °C for 15 min, and protein concentrations were determined with a BCA protein assay kit (Beyotime, Shanghai, China). Subsequently, equal amounts of protein samples were separated by electrophoresis using 6, 10 or 12.5% SDS-PAGE Gel Fast Preparation Kits (Epizyme, Shanghai, China) and then transferred to polyvinylidene difluoride (PVDF) membranes. The membranes were incubated with antibodies against the following proteins: β-actin (1:1000; Proteintech), Akt (1:1000; Proteintech), phospho-Akt (Ser473) (1:1000; CST), TGM2 (1:1000; CST), Kelch-like ECH-associated protein 1 (KEAP1) (1:1000; Proteintech), NF-E2-related factor 2 (NRF2) (1:1000; Proteintech), mTOR (1:1000; Proteintech), phospho-mTOR (S2448) (1:1000; Abcam), Bax (1:1000; Proteintech), Bcl2 (1:1000; Proteintech), Caspase 3 (1:500; Proteintech), PARP1 (1:1000; Proteintech), and cleaved PARP1 (1:1000; Abcam).

### RNA preparation and quantitative real-time PCR

Total RNA was extracted with RNAiso Plus (Takara, Tokyo, Japan). Reverse transcription was conducted using Takara PrimeScript™ RT Master Mix to obtain cDNA. Quantitative RT-PCR was performed with TB Green® Premix Ex Taq™ to determine the expression of candidate genes using an ABI 7900HT Real-Time PCR system (Applied Biosystems, CA, USA). The primer sequences are listed in Table 1.

**Table 1:** Primer sequences performed in the text

TGM2 Forward	5′-GAGGAGCGGCAGGAGTATG- 3′
TGM2 Reverse	5′-CAGGAACTTGGGGTTGACATC- 3′
β-actin Forward	5′-TTGTTACAGGAAGTCCCTTGCC- 3′
β-actin Reverse	5′-ATGCTATCACCTCCCCTGTGTG- 3’

### mRNA sequencing

Total RNA was isolated from pancreatic cells treated with kaempferol (50 μM) and control cells were isolated with TRIzol reagent (Invitrogen, China). Both cell lines were analyzed in triplicate by mRNA sequencing, which was conducted by Sangon Bitotech (Shanghai, China) using the Dual-mode mRNA Library Prep Kit for Illumina. The fragments per kilobase of exon per million fragments mapped reads (FPKM) for each gene were calculated after data processing [18].

### Reactive oxygen species detection

The accumulation of intracellular ROS was assessed using an ROS assay kit (Beyotime, Shanghai, China). Cells were incubated with 10 μmol/L DCFH-DA at 37 °C for 30 min before being collected and suspended in ice-cold PBS. DCFH-DA is further oxidized by ROS after deacetylation to form 2,7-dichlorofluorescein (DCF). The DCF fluorescence intensities were then measured by flow cytometry at an excitation wavelength of 488 nm and an emission wavelength of 525 nm. ROS levels in TGM2-overexpressing cell lines were detected using dihydroethidium (DHE), which avoids the effects of EGFP fluorescence.

### Immunohistochemistry staining

Immunohistochemistry (IHC) staining was performed as previously described [19]. In brief, the paraffin-embedded tumor sections were dewaxed in xylene and then rehydrated with a graded alcohol series. A 3% H_2_O_2_ methanol buffer was used to block endogenous peroxidase activity, and citrate buffer (pH 6.0) was used for antigen retrieval. Tumor sections were incubated with a primary anti-TGM2 antibody (1:300, CST) at 4 °C overnight and then incubated with a secondary antibody at room temperature. TGM2 expression levels were assessed by determining the immunoreactive scores (IRS), which were determined by multiplying the percentage of positive cells ((0, < 5%; 1, 5 ~ 25%; 2, 25 ~ 50%; 3, 50% ~ 75%; and 4, 75% ~ 100%) by the intensity scores (0, negative expression; 1, weak; 2, moderate; 3, strong). A score ≥ 4 was defined as high TGM2 expression, with lower scores indicating low expression.

### In vivo tumor Xenograft models

Twelve male BALB/c mice (4–6 weeks old, 18–20 g) were purchased from Shanghai Jihui Experimental Animal Breeding Co., Ltd. and housed under specific pathogen-free conditions with free access to food and water. Approximately 1 × 10^7^ PANC-1 cells in 200 μl of PBS were subcutaneously injected into the right flank of each mouse to establish the tumor model. The mice with tumor xenografts were then randomly assigned to the kaempferol and control groups (*n* = 6 in each group). Treatment was initiated when a tumor reached approximately 100 mm^3^ in size. Many previous experiments have confirmed that an appropriate high dose of kaempferol does not produce cytotoxicity but plays a protective role. For instance, Cheng X et al. suggested that the pretreatment with kaempferol (25, 50 and 100 mg/kg) by gavage for 7 days significantly alleviated inflammatory responses and blood-brain barrier dysfunction [20]. Luo C et al. showed that kaempferol at doses of 50 and 150 mg/kg dose-dependently improved blood lipids in the treatment of diabetic rats [21]. Based on the abovementioned dose safety report, we chose a dose of 100 mg/kg for intragastric administration. The mice in the control and kaempferol-treated groups were orally administered PBS or 100 mg/kg kaempferol for 2 weeks daily, respectively. The mice were closely monitored starting from the first treatment, and the tumor sizes were calculated twice every week using the following formula: tumor volume = length × (width)^2^/2. The mice were sacrificed 6 weeks after injection, and paraffin-embedded tumor tissue sections were generated for subsequent analysis.

### Statistical analysis

Experiments were repeated at least three times, and the results are presented as the means ± SD. Two-tailed unpaired Student’s t tests or one-way ANOVA were used to evaluate the data. GraphPad Prism (version 7.0a) was used for the data analysis. Differences were considered statistically significant at *P* < 0.05.

## Results

### Cytotoxic effects of kaempferol on pancreatic cancer

**Fig. 1:**
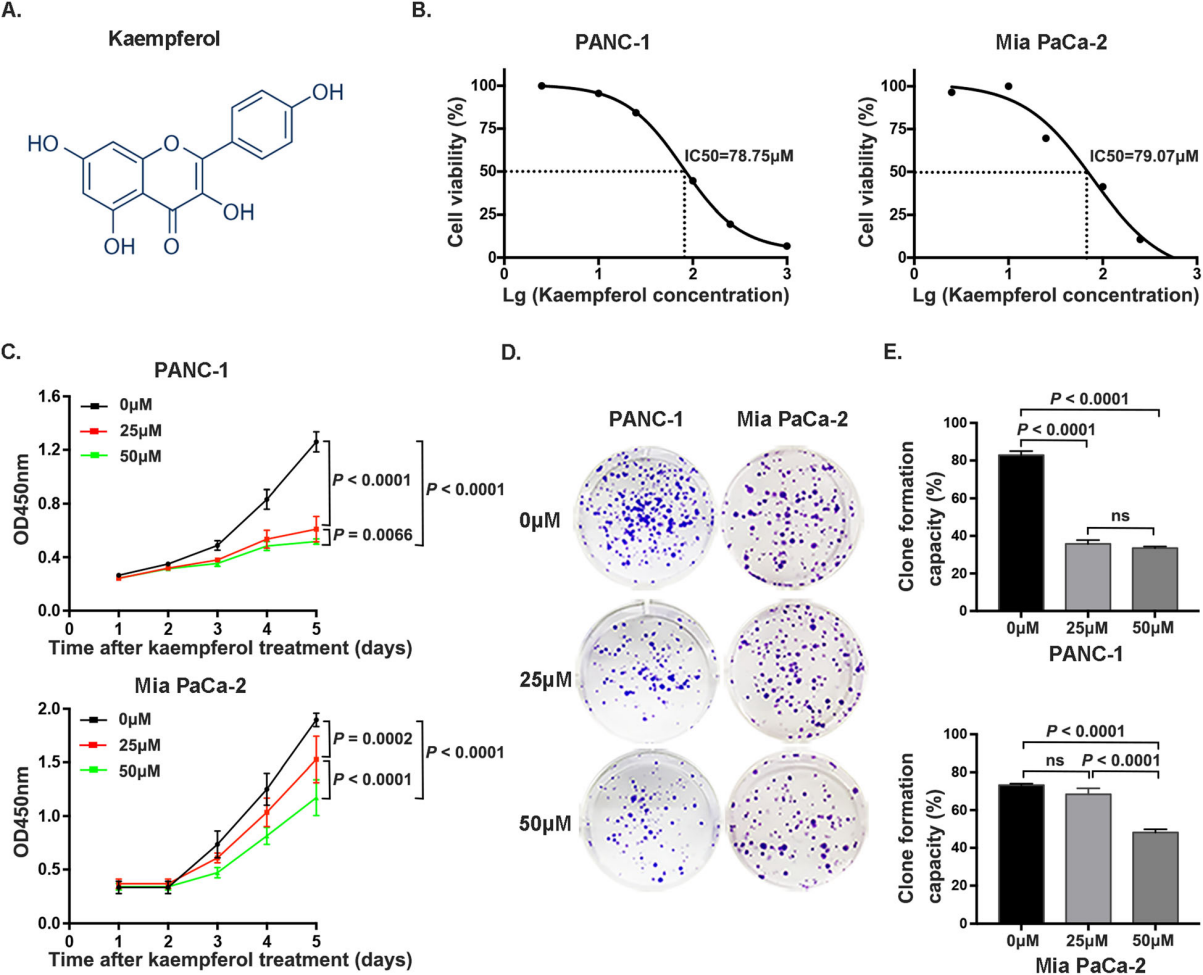
Kaempferol suppresses pancreatic cancer cell proliferation and formation capacity. **a** The chemical structure of kaempferol. **b** The cytotoxicity of kaempferol at various concentrations (0 to 1000 μM) in PANC-1 and Mia PaCa-2 cells, which showed IC50 values of 78.75 and 79.07 μM, respectively. **c** Dose-dependent inhibitory effect of kaempferol (0, 25 and 50 μM) on pancreatic cancer cell growth. **d** and **e** Kaempferol inhibits the colony formation capacity of PANC-1 and Mia PaCa-2 cells. A *P* value less than 0.05 was considered statistically significant

Kaempferol is a flavonoid compound that is primarily found in the Chinese herbal medicine Zingiberaceae, and its structure is shown in Fig. 1a. Kaempferol exhibited anticancer effects on PANC-1 and Mia PaCa-2 cells in a concentration-dependent manner, with decreased cell viability observed at high concentrations. The IC50 values of kaempferol in PANC-1 and Mia PaCa-2 cells were 78.75 μM and 79.07 μM, respectively (Fig. 1b). Based on the observed IC50 values, kaempferol was used at concentrations of 0, 25 and 50 μM in subsequent analyses. The CCK-8 assay results showed that the rates of cell proliferation were inhibited after 24 h, and significant dose-dependent inhibitory effects between 25 μM and 50 μM were detected after 96 h in both PANC-1 and Mia PaCa-2 cells (*P* < 0.05) (Fig. 1c). Furthermore, colony formation assay results indicated that colony formation was suppressed by treatment with increasing concentrations of kaempferol (Fig. 1d, e).

### Kaempferol induces apoptosis through the Akt/mTOR signaling pathway

**Fig. 2:**
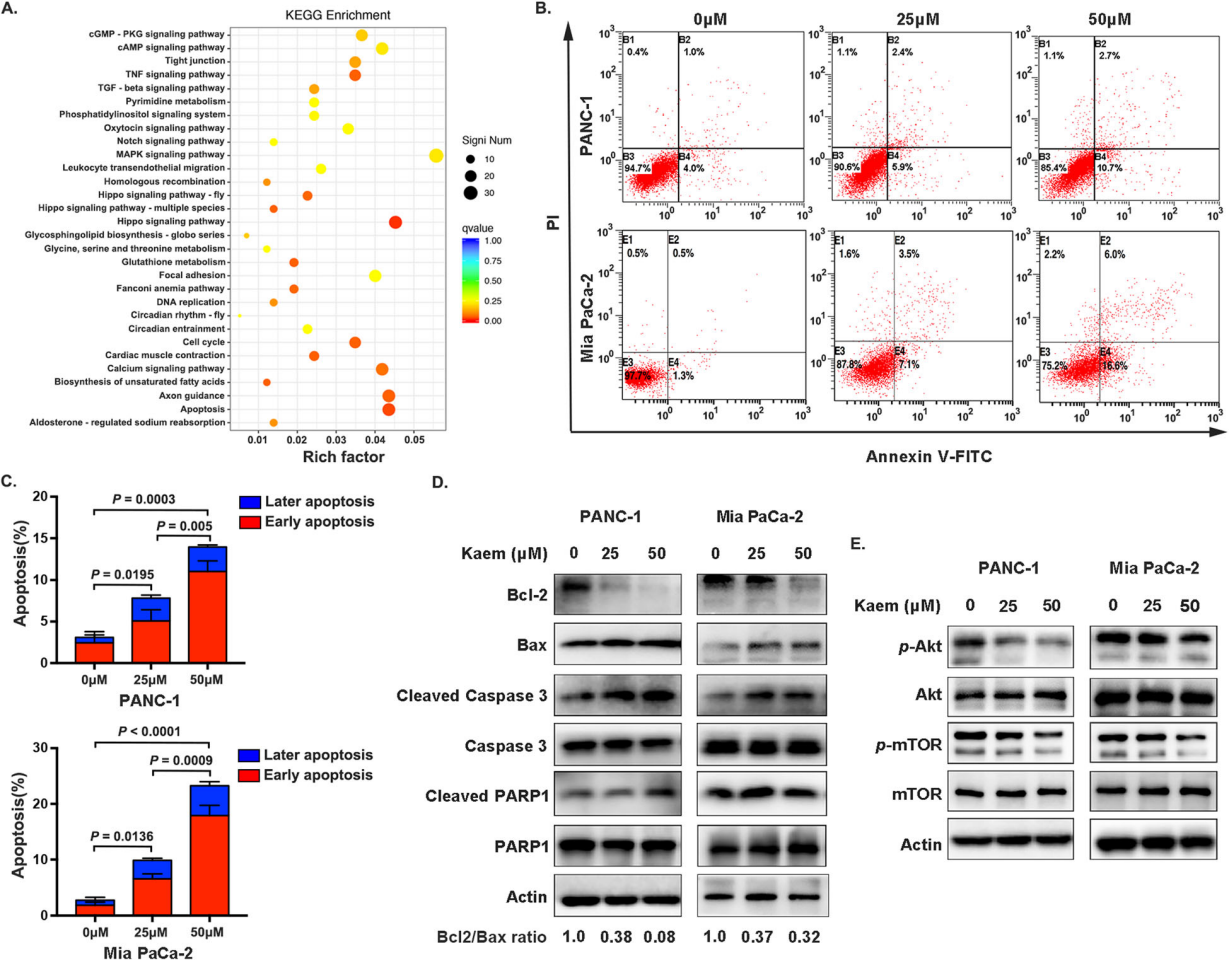
Kaempferol induces pancreatic cancer cell apoptosis. **a** KEGG pathway enrichment analysis of differentially expressed genes after treatment with 50 μM kaempferol. **b** and **c** Kaempferol increased the apoptosis in PANC-1 and Mia PaCa-2 cells (*P* < 0.05). **d** Representative western blotting images of apoptotic phenotype markers. The ratio of Bcl2 to Bax was determined using ImageJ software. The grouping of blots cropped from different parts of the same gel, or from different gels, fields, or exposures was divided with white space. **e** Representative western blotting images of downregulated phosphorylation of Akt and mTOR after treatment with kaempferol. The grouping of blots cropped from different parts of the same gel or from different gels, fields, or exposures was divided with white space

RNA sequencing was performed to investigate the underlying mechanisms by which kaempferol inhibits pancreatic cancer. KEGG enrichment analysis based on significantly differentially expressed genes revealed a series of enriched signaling pathways, including apoptosis signaling (Fig. 2a). To further confirm these results, cells were examined using flow cytometry after incubation with 0, 25 and 50 μM kaempferol for 48 h. Our results showed a significant increase in PANC-1 and Mia PaCa-2 cell apoptosis in response to 50 μM of kaempferol (13.93 ± 1.474% and 21.50 ± 3.195%, respectively), and the rate of apoptosis was positively correlated with kaempferol concentration (Fig. 2b, c). Moreover, this finding is consistent with the observed levels of important proteins involved in cell apoptosis, such as Bcl-2, Bax, Caspase 3, and PARP1 (Fig. 2d). The Akt/mTOR signaling pathway, which is widely acknowledged to have crucial functions in cells, is activated in many cancers and inhibits apoptosis, contributing to drug resistance in the clinic [22]. Consistent with these previous findings, in our present study, Akt and mTOR phosphorylation were downregulated after 48 h of kaempferol treatment (Fig. 2e). Collectively, our results indicate that Akt/mTOR signaling inactivation is involved in kaempferol-induced apoptosis of pancreatic cells.

### Kaempferol-mediated Akt/mTOR signaling occurs in an ROS-dependent manner

**Fig. 3:**
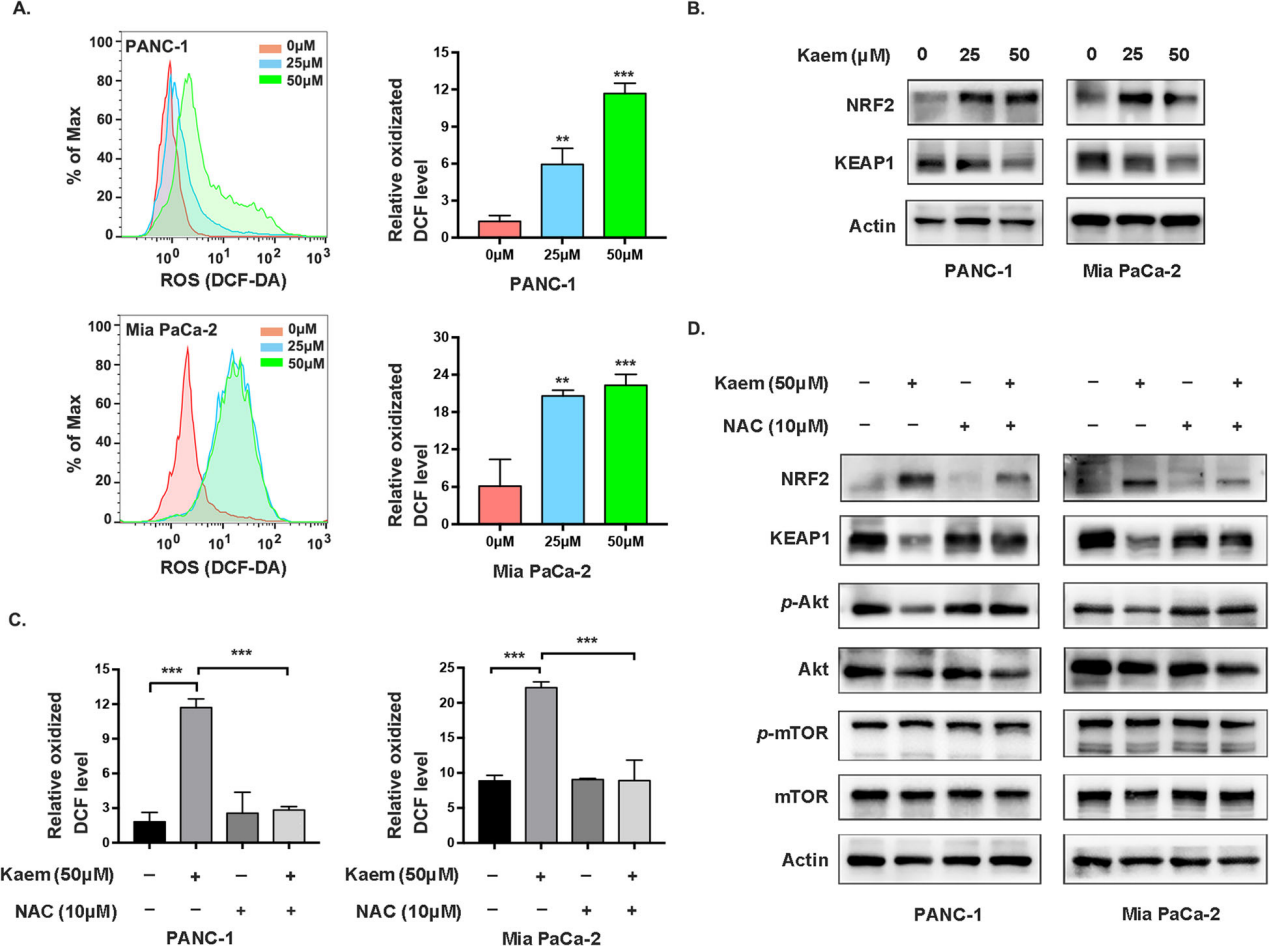
Kaempferol regulates Akt/mTOR inactivation in a ROS-dependent manner. **a** Left, ROS levels in PANC-1 and Mia PaCa-2 cells treated with kaempferol were detected by flow cytometry; right, quantification of the mean ROS production, which is presented as the means ± SD (**P < 0.01, ***P < 0.001). **b** Kaempferol treatment increased NRF2 protein levels and decreased those of KEAP1. The grouping of blots cropped from different parts of the same gel or from different gels, fields, or exposures was divided with white space. **c** Increased ROS levels in cells treated with kaempferol were suppressed by 10 mmol/L NAC. **d** After treatment with 10 mmol/L NAC for 2 h, NRF2 protein levels were decreased, while those of KEAP1 were increased. Akt phosphorylation at Ser473 was increased in PANC-1 and Mia PaCa-2 cells. The grouping of blots cropped from different parts of the same gel or from different gels, fields, or exposures was divided with white space

Kaempferol, a flavonoid phytochemical, has a potential role in regulating cellular processes by mediating oxidative stress [23, 24]. Therefore, we hypothesized that kaempferol disrupts Akt/mTOR signaling by inducing alterations in ROS levels. Therefore, we assessed the production of ROS after treatment with various concentrations of kaempferol using an ROS assay kit. ROS levels increased in cancer cells concomitantly with increases in the kaempferol concentration (Fig. 3a). The KEAP1-NRF2 system is the major node that senses and responds to redox disturbances resulting from intrinsic and extrinsic factors [25, 26]. The dual roles of the KEAP1-NRF2 pathway in preventing or initiating pancreatic cancer primarily depend on the stages of cancer and the states of KEAP1 and NRF2 [27]. Therefore, in our present study, we subsequently evaluated KEAP1 and NRF2 expression to confirm the occurrence of redox imbalance. Immunoblotting results indicated that KEAP1 levels were downregulated, while NRF2 levels were increased to some extent in pancreatic cancer cells treated with kaempferol (Fig. 3b). Subsequently, we examined the effects of NAC, which scavenges ROS, on Akt and mTOR phosphorylation, and diminished ROS levels are shown in Fig. 3c. The decrease in Akt Ser473 and mTOR S2448 phosphorylation in PANC-1 and Mia PaCa-2 cells was reversed by NAC treatment (Fig. 3d). In summary, our results demonstrated that kaempferol increases ROS levels, which in turn suppresses the Akt/mTOR signaling pathway.

### Kaempferol downregulates TGM2 in vitro and in vivo, and TGM2 expression is negatively correlated with PDAC prognosis

**Fig. 4:**
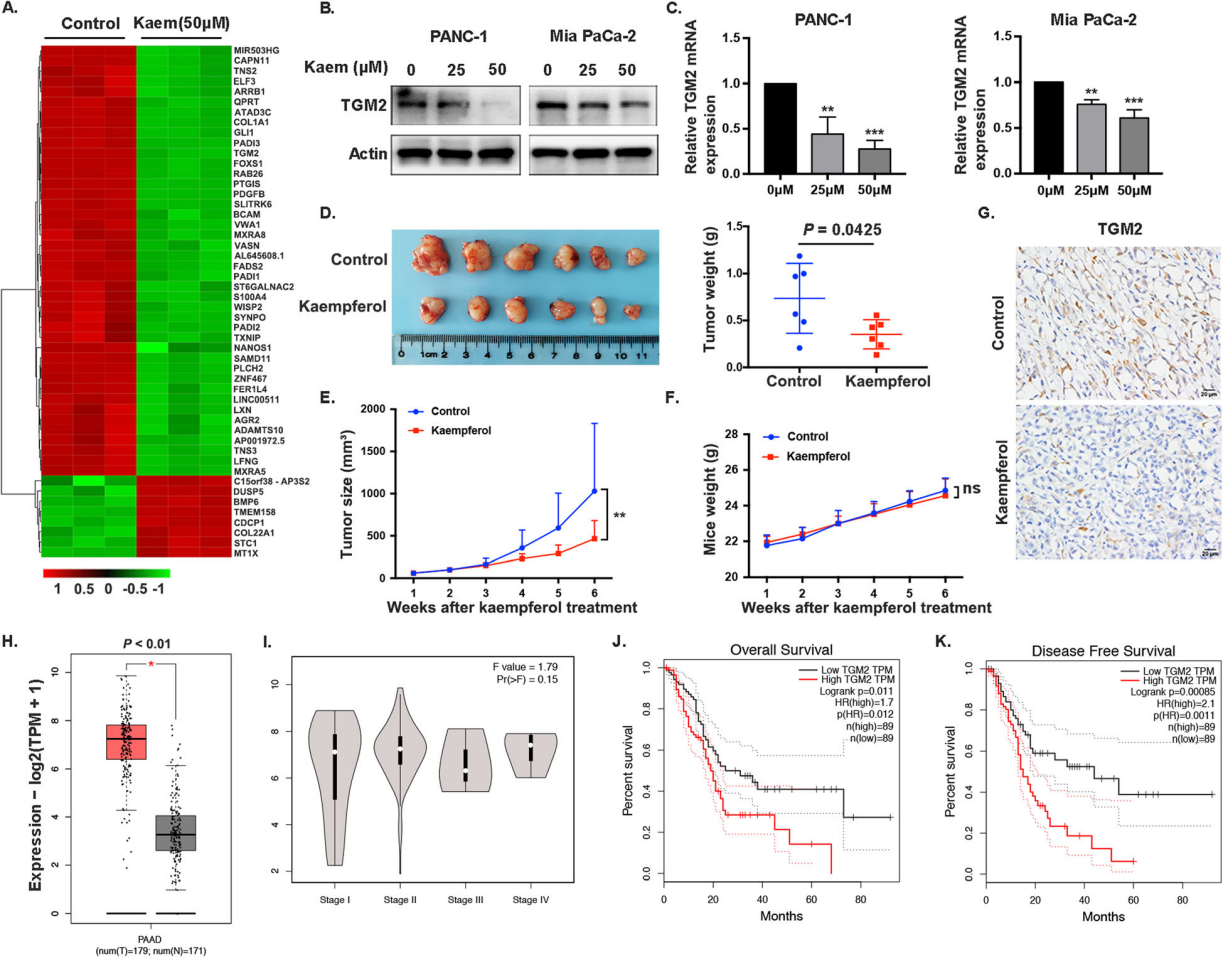
Kaempferol downregulation of TGM2 and TGM2 expression is negatively correlated with PDAC prognosis. **a** Heatmap showing the clustering of the top 50 differentially expressed genes in PANC-1 cells treated with kaempferol (50 μM) and control cells. **b** and **c** TGM2 protein levels and mRNA levels in PANC-1 and Mia PaCa-2 cells were decreased by kaempferol treatment compared with those observed in control cells (**P < 0.01, ***P < 0.001). The grouping of blots cropped from different parts of the same gel or from different gels, fields, or exposures was divided with white space. **d** Left, antitumor activity of kaempferol (100 mg/kg) in nude mice; right, tumor weight. **e** Tumor sizes were calculated using the following formula: tumor volume = length × (width)2 /2 (*P* = 0.005). **f** Total mice weight. **g** TGM2 expression in xenograft tissue sections was examined by IHC (scale bar, 20 μm). **h** TGM2 transcription levels were significantly higher in pancreatic cancer tissues than in normal tissues from the GEPIA database (*P* < 0.01). **i** TGM2 transcription at different stages (*P* = 0.15). **j** and **k** High TGM2 mRNA expression was correlated with shorter OS (*P* = 0.011) and DFS (*P* = 0.000)

The results of a heat map clustering analysis revealed notable differences in gene expression between the control and kaempferol-treated groups, with significant TGM2 downregulation detected in the kaempferol-treated groups (Fig. 4a). Decreased TGM2 mRNA and protein levels were observed in the cells after treatment with kaempferol (Fig. 4b, c). In addition, we assessed the effect of kaempferol on tumor growth in vivo using a subcutaneous xenograft model. Mice treated with 100 mg/kg of kaempferol exhibited significantly suppressed the tumor growth, including tumor weight and tumor volume, compared to that observed in the control group (Fig. 4d, e). Furthermore, kaempferol had no significant inhibitory effect on the weights of mice (Fig. 4f). To further investigate the role of TGM2 in pancreatic cancer, we performed IHC staining to examine TGM2 expression in tumor tissues (Fig. 4g). The results showed that TGM2 levels were significantly decreased in the group treated with kaempferol, which was consistent with the in vitro findings. Subsequently, we analyzed publicly assessable data using the gene expression profiling interactive analysis (GEPIA) tools to assess TGM2 gene expression in PDAC and normal tissues (number (T) = 179 and number (N) = 171, respectively). The results showed higher TGM2 transcription in PDAC tissues than in normal tissues, while differences were not observed between the different stages (Fig. 4h, i). Furthermore, higher TGM2 mRNA expression was correlated with shorter overall survival (OS) (*P* = 0.011) and disease-free survival (DFS) (*P* = 0.000) (Fig. 4j, k). These results indicated that TGM2 may be a potential target of kaempferol to inhibit pancreatic cancer and could serve as a promising prognostic biomarker for this disease.

### TGM2 functions as a target of kaempferol to promote pancreatic cancer cell apoptosis via ROS-dependent Akt/mTOR signaling

**Fig. 5:**
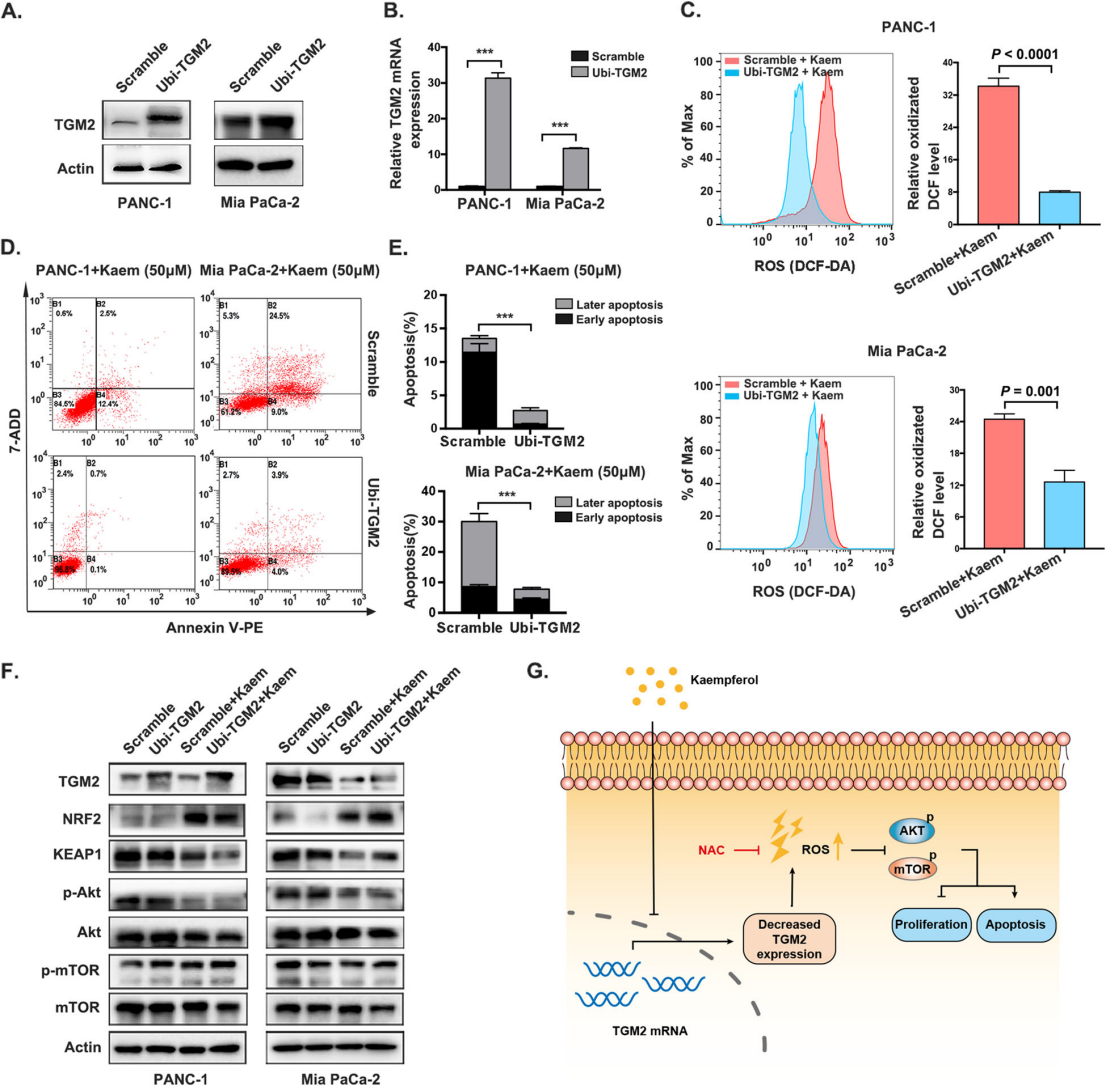
TGM2 functions as a target of kaempferol to induce pancreatic cancer cell apoptosis. **a** and **b** TGM2 protein and mRNA overexpression in PANC-1 and Mia PaCa-2 cells was confirmed by immunoblotting analysis and quantitative RT-PCR, respectively (***P < 0.001). The grouping of blots cropped from different parts of the same gel or from different gels, fields, or exposures was divided with white space. **c** Left, ROS alterations in TGM2-overexpressing PANC-1 and Mia PaCa-2 cells treated with kaempferol; right, quantification of ROS production in both TGM2-overexpressing pancreatic cancer cell lines, which is presented as the means ± SD (*P* < 0.0001 and *P* = 0.001, respectively). **d** and **e** Apoptotic rates of the TGM2-overexpressing and TGM2-scramble cell lines following kaempferol treatment (***P < 0.001). **f** Immunoblotting analysis of TGM2, NRF2, KEAP1, p-Akt, and p-mTOR levels in PANC-1 and Mia PaCa-2 cells overexpressing TGM2 following kaempferol treatment. The grouping of blots cropped from different parts of the same gel or from different gels, fields, or exposures was divided with white space. **g** A schematic illustrating the proposed ROS-associated mechanism by which kaempferol downregulates TGM2 expression to increase ROS levels and promote Akt/mTOR signaling inactivation, which contributes to the inhibition of cell proliferation and the induction of apoptosis

Given the crucial role of TGM2 in responding to oxidative stress, which involves various cellular processes, including apoptosis, we next investigated the potential functions of TGM2 in pancreatic cancer. We hypothesized that TGM2 serves as a target of kaempferol to facilitate apoptosis, which may be correlated with ROS-dependent Akt/mTOR signaling in pancreatic cancer. To assess this possibility, we stably overexpressed TGM2 in PANC-1 and Mia PaCa-2 cells, and the overexpression efficiency was confirmed by western blotting and quantitative RT-PCR (Fig. 5a, b). ROS production was significantly decreased upon overexpression of TGM2 in both the pancreatic cancer cell lines (Fig. 5c). The rate of cell apoptosis was accordingly reversed in TGM2-overexpressing PANC-1 and Mia PaCa-2 cells in response to 50 μM kaempferol treatment when compared to the scramble group (2.733 ± 0.285% vs 16.8 ± 1.044% and 7.733 ± 0.328% vs 30.03 ± 1.749%, respectively) (Fig. 5d, e), suggesting that TGM2 plays a crucial role in pancreatic cancer cell apoptosis. Moreover, the observed levels of KEAP1, NRF2, p-AKT and p-mTOR in TGM2-overexpressing cells following kaempferol treatment were consistent with the aforementioned results, indicating that kaempferol promotes apoptosis via the TGM2-mediated ROS-dependent Akt/mTOR signaling pathway (Fig. 5f).

## Discussion

Redox biology is ubiquitous among different cell types and biological systems and is important in many different fields of study, such as neuroscience, inflammation and cancer [28]. Redox homeostasis is essential for aerobic systems and is dependent on a sustained balance between ROS formation and detoxification [29]. It has long been postulated that tumor cells exhibit continuous and elevated ROS production as a consequence of genetic, metabolic and microenvironment-associated alterations [29]. Interestingly, it seems paradoxical that cancer cells reprogram metabolic mechanisms toward glutamine utilization in the TCA cycle to attenuate ROS-induced cell damage and maintain cellular redox homeostasis [30]. Considering their abovementioned dualistic nature, ROS act as harmful or beneficial molecules to regulate cellular physiology or trigger cytotoxicity depending on their duration and concentration [31]. Therefore, the development of strategies aimed at leveraging cellular redox signaling events in cancer to develop effective anticancer therapeutic treatments or targeting ROS regulators is important for cancer treatment.

As one of the processes associated with cancer initiation and development, apoptosis is a major type of programmed cell death (PCD) that invariably determines the fate of malignant cells to some extent [32]. Furthermore, the Akt/mTOR cascade has emerged as a major regulator in cancer cell apoptosis [33]. Fiorini, C. et al. demonstrated that elevated ROS can act as adaptors to promote pancreatic cancer chemosensitivity and reduce autophagy by inducing the Akt/mTOR pathway [34]. Of note, whether the upregulation or scavenging of ROS in pancreatic cancer involves intricate interrelationships among factors involved in Akt/mTOR-induced apoptosis remains controversial.

Traditional Chinese medicine (TCM) treatments have drawn close attention because of their diverse pharmacological activities, such as anti-inflammatory, antitumor and antioxidant activities, high efficacy and low toxicity [35]. Kaempferol, a typical flavonol-type flavonoid that is abundantly found in a variety of Chinese herbs, exhibits strong inhibitory activity toward cancers, such as gastric cancer and lung cancer [36, 37]. In recent years, kaempferol has become known for its potent ability to scavenge free radicals and superoxide radicals as well as its ability to activate antioxidant enzymes [38]. However, an increasing number of studies have recently focused on its ability to inhibit cancer by triggering ROS production [14, 39]. In the present study, we observed that when cell apoptosis occurred, ROS levels increased, NRF2 proteins were activated, and KEAP1 expression was downregulated. In the past, NRF2 was proven to contribute to tumor progression and was associated with poor prognosis [40, 41]. However, accumulating evidence consistent with our work suggests that the activation of NRF2 is able to inhibit tumorigenesis. For instance, multiple compounds, such as sulforaphane and curcumin, have been discovered to exert anticancer activities by activating NRF2/ARE-regulated genes, such as hemeoxygenase-1 (HO-1) and NAD(P)H quinone oxidoreductase 1 (NQO1) [42]. However, the precise mechanism by which kaempferol activates NRF2 to affect apoptosis in pancreatic cancer needs to be further investigated in our subsequent work.

Mammalian transglutaminases (TGs) are a family of structurally and functionally related proteins with enzymatic and scaffolding functions that participate in the regulation of cell processes and are implicated in the development of disease [43, 44]. TGM2 was the first TG member to be discovered and is a multifunctional protein involved in the pathogenesis of multiple types of cancers [45, 46]. It has been reported to inhibit apoptosis and is associated with chemotherapy resistance in colorectal cancer (CRC) via activation of Wnt/β-catenin signaling [47]. In our present study, we showed that the level of TGM2 transcription was significantly higher in PDAC tissues than in normal tissues. Furthermore, increased TGM2 mRNA expression was correlated with shorter OS and DFS, suggesting that TGM2 may serve as a prognostic biomarker for pancreatic cancer. Intriguingly, TGM2 is known to be a stress-response gene that can be activated by various stressors, including inflammatory cytokines and ROS [48]. In the present study, our findings showed that kaempferol treatment caused the pharmacological inhibition of TGM2 in vitro and in vivo, and a negative correlation was observed between TGM2 expression and the regulation of ROS-dependent apoptosis, indicating that TGM2 may be a potential anticancer target of kaempferol for pancreatic cancer treatment.

## Conclusions

In summary, the results of our present study indicate that kaempferol induces ROS-dependent apoptosis in pancreatic cancer cells via TGM2-mediated Akt/mTOR signaling (Fig. 5g), and TGM2 may represent a promising prognostic biomarker for pancreatic cancer.

## Data Availability

All data generated or analyzed during this study are included in this published article. RNA-sequencing data have been deposited into the NCBI Sequence Read Archive (SRA) database (https://www.ncbi.nlm.nih.gov/sra) under accession number PRJNA689118.

## Abbreviations

ROS: reactive oxygen species
GEPIA: Gene Expression Profiling Interactive Analysis
TGM2: tissue transglutaminase
NAC: N-acetyl-L-cysteine
PIG3: p53-inducible gene 3
TG: Transglutaminase
CRC: Colorectal cancer
PDAC: Pancreatic ductal adenocarcinoma
ATCC: American Type Culture Collection
DMEM: Dulbecco’s modified Eagle’s medium
DMSO: Dimethyl sulfoxide
CCK-8: Cell Counting Kit-8
IC50: The half maximal inhibitory concentration
PI: Propidium iodide
PBS: Phosphate-buffered saline
PMSF: Phenylmethanesulfonyl fluoride
PVDF: Polyvinylidene difluoride
KEAP1: Kelch-like ECH-associated protein 1
NRF2: NF-E2-related factor 2
RT-PCR: Real-Time PCR
DCF: 2,7-dichlorofluorescein
DHE: Dihydroethidium
LSCM: Laser scanning confocal microscopy
TCM: Traditional Chinese medicine
PCD: Programmed cell death
